# Abdominal aortic aneurysms part two: Surgical management, postoperative complications and surveillance

**DOI:** 10.1177/1750458920947352

**Published:** 2020-09-08

**Authors:** Harry Kyriacou, Ahmed M H A M Mostafa, Anoop S Sumal, Holly N Hellawell, Jonathan R Boyle

**Affiliations:** 1 University of Cambridge School of Clinical Medicine, Addenbrooke’s Hospital, Cambridge, UK; 2 Cambridge University Hospitals, NHS Foundation Trust, Cambridge Vascular Unit, Cambridge, Cambridgeshire, UK

**Keywords:** Abdominal aortic aneurysm, Cardiovascular disease, Complications, Surveillance, Vascular surgery

## Abstract

Large, symptomatic and ruptured abdominal aortic aneurysms are usually treated surgically if patients are deemed fit enough. This may be achieved through endovascular or open surgical repair. The type of treatment that a patient receives is dependant on many factors, such as the rupture status of the aneurysm. Each approach is also associated with different risks and postoperative complications. Multiple guidelines exist to inform the surgical management of abdominal aortic aneurysms. This literature review combines these recommendations and explores the evidence upon which they are based. In addition, it highlights the key perioperative considerations that need to be considered in cases of unruptured and ruptured abdominal aortic aneurysms.

**Provenance and Peer review:** Unsolicited contribution; Peer reviewed; Accepted for publication 5 July 2020.

## Introduction

An abdominal aortic aneurysm (AAA) is defined as an irreversible dilatation of the abdominal aorta to a diameter greater than 3.0 cm or 1.5 times its normal anteroposterior diameter (NICE 2020). There are many risk factors that contribute to AAA development, including age, male sex and smoking status. As a result, many countries including the United Kingdom (UK) have developed screening programmes for at-risk groups. In the UK, AAA screening is offered to men within the year of their sixty-fifth year birthday (Public Health England 2019). In 2019, this identified AAAs in approximately 1% of all men screened (Public Health England 2019).

However, not all AAAs are asymptomatic; patients with an unruptured AAA may experience a persistent abdominal or lower back pain and a pulsating sensation in the abdomen, while those with a ruptured abdominal aortic aneurysm (rAAA) may present with sudden, severe back pain and shock (NHS 2017). Unfortunately, rAAAs are the primary presentation for 50% of patients and have a mean in-hospital mortality rate of 35.4% after repair (Jeanmonod et al 2020; National Vascular Registry 2019).

Due to the poor outcomes associated with rAAAs, the National Institute for Health and Care Excellence (NICE), European Society for Vascular Surgery (ESVS) and the Society for Vascular Surgery (SVS) have published comprehensive guidelines on the surgical management and surveillance of patients with AAAs (Chaikof et al 2018, NICE 2020, Wanhainen et al 2019). This review explores these recommendations, highlighting the key perioperative factors that need to be considered in the care of these patients.

## Decision for surgery

According to NICE, surgical repair should be considered for unruptured AAAs in patients that are either symptomatic, asymptomatic with an AAA >4 cm that has grown >1 cm in one year, or asymptomatic with an AAA ≥5.5 cm (NICE 2020). Similarly, the ESVS guidelines recommend surgery for male patients with AAA diameters of ≥5.5 cm (Wanhainen et al 2019). In comparison, surgery may be considered for female patients with AAA diameters of ≥5.0 cm, or between 5 and 5.4 cm (Chaikof et al 2018; Wanhainen et al 2019). The decision to treat must also consider other factors, such as patient preference and fitness for surgery (NICE 2020). Currently, the ESVS guidelines do not recommend elective repair of AAA in those with limited life expectancy (Wanhainen et al 2019). rAAAs, however, are a true emergency and require urgent repair in patients fit for surgery (Chaikof et al 2018). Both unruptured AAAs and rAAAs can be treated using endovascular aneurysm repair (EVAR) or open surgical repair (OSR).

### Unruptured AAAs

#### EVAR

**Figure 1:**
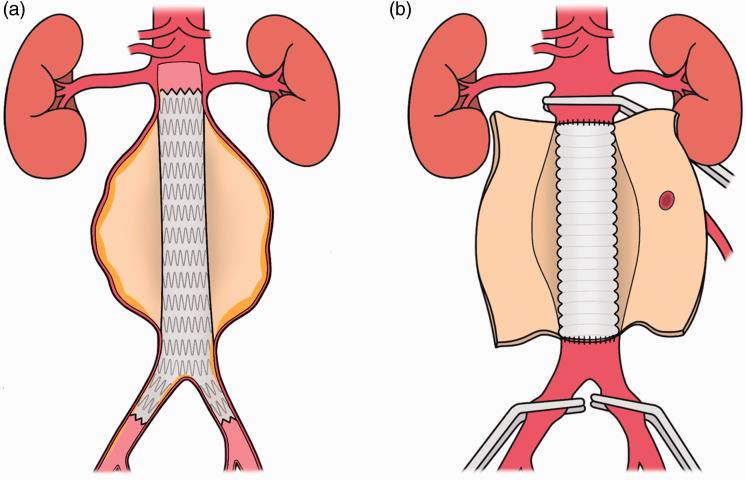
Surgical repair of AAAs. (a) Cross section of an AAA treated via EVAR. (b) OSR

EVAR is a minimally invasive, X-ray-guided method of AAA repair that involves inserting an expandable stent (endograft), into the AAA through an incision in the groin (Figure 1(a)). This patches the aneurysm and reduces the risk of rupture. While EVAR does not result in a lower overall mortality in elective AAA repair, it is associated with decreased operative time, blood loss, total hospital length of stay and postoperative pulmonary complications, compared to OSR (Paravastu et al 2014). As a result, EVAR is now the most common technique for elective repair in the UK (Johal et al 2019). It should be noted, however, that elective EVAR has recently decreased in the UK due to the publication of the draft NICE guidelines, which discouraged its use (Loftus et al 2020).

While NICE acknowledges the superiority of EVAR in the short term, they only recommend EVAR for unruptured AAAs in patients who meet the aforementioned criteria *and* are contraindicated from OSR (NICE 2020). Reasons for this include a lack of benefit in long-term survival, more long-term complications and re-interventions and a lack of cost-effectiveness (NICE 2020). The ESVS makes a more relaxed recommendation, that EVAR should be used first in those with suitable anatomy and reasonable life expectancy (Wanhainen et al 2019). If a patient has low life expectancy, elective AAA repair is not recommended (Wanhainen et al 2019).

#### OSR

During OSR, an abdominal incision is made to reach the AAA, which is then replaced with a durable prosthetic graft (Figure 1(b)). OSR involves clamping the abdominal aorta, which can lead to significantly higher rates of acute kidney injury (AKI) than EVAR after surgery (Ambler et al 2015). Advantages of OSR include a lower rate of long-term complications over a mean follow-up of 12.7 years and fewer re-interventions compared to EVAR (12% vs. 26%) (Patel et al 2016).

NICE recommends OSR for patients with unruptured AAAs who meet the above criteria and do not have the following contraindications: abdominal co-pathology, high anaesthetic risk and medical comorbidities (NICE 2020). NICE have not precisely defined medical comorbidities or high-anaesthetic risk, so clinicians have some degree of flexibility in interpreting this (Loftus et al 2020). The SVS notes that OSR is used in patients who do not meet the anatomical requirements for EVAR (Chaikof et al 2018). These include those with severe aortic angulation, excessive thrombus or even the presence of numerous accessory renal arteries (Chaikof et al 2018). The ESVS guidelines, however, recommend OSR over EVAR in all patients with long life expectancy (Wanhainen et al 2019).

##### Mortality rates

While the overall mortality rates are similar between EVAR and OSR, they differ at various time points after surgery (Patel et al 2016). Patients undergoing EVAR have a significantly lower 30-day or in-hospital mortality rate than OSR patients (1.3% vs. 4.7%), which continues for the first six months (4% vs. 7%) (Patel et al 2016; Stather et al 2013). This early advantage is corroborated by more recent data, in which the in-hospital mortality rate for infra-renal AAAs is reported as 0.4% after EVAR, compared with 3.2% after OSR (National Vascular Registry 2019). However, between six months and four years, there is no significant difference in mortality between methods (21% vs. 20%) (Patel et al 2016). In fact, due to the high rate of long-term complications associated with EVAR, the mortality rate >8 years post-intervention is significantly greater compared to OSR (53% vs. 46%) (Patel et al 2016).

### Ruptured AAAs

rAAAs treated with EVAR have been shown to have a lower in-hospital postoperative mortality rate than OSR (22.6% vs. 40.9%) (National Vascular Registry 2019). For the treatment of rAAAs, NICE note that EVAR provides more benefit than OSR for most people, especially men over 70 (NICE 2020). The SVS also advocates the use of EVAR over OSR for rAAAs if it is anatomically feasible (Chaikof et al 2018). The ESVS notes that a particular advantage of EVARs in cases of rAAA is the ability to perform the procedure under local anaesthesia, which, if used alone, results in a reduced 30-day mortality (Wanhainen et al 2019). Contrastingly, OSR provides a better balance of benefits and harms in men under 70 and should also be considered in cases where standard EVAR is unsuitable (NICE 2020). For women, NICE recommends EVAR at any age (NICE 2020). In fact, women have been shown to benefit more from EVAR compared to men (Powell et al 2014). Ultimately, irrespective of the method chosen, the SVS recommends a door-to-intervention time of <90 min for rAAAs (Chaikof et al 2018).

## Postoperative considerations

### Location of care

The decision to admit patients following AAA repair should be made on a case-by-case basis, depending on their stability, risk factors and intraoperative complications. For example, ICU admission is advisable in patients who require mechanical ventilation and those that had haemodynamic instability during surgery (Chaikof et al 2018). Unnecessary admission occupies valuable bed space and according to a recent report, an ICU stay after OSR is associated with higher rates of atrial fibrillation, pneumonia, kidney injury and five-year mortality compared to discharge to a vascular surgery ward (Varetto et al 2019). As such, the SVS recommends that only patients who have pre-existing cardiac, pulmonary and/or renal conditions should be routinely managed in an ICU after surgery (Chaikof et al 2018). Wherever these patients are ultimately managed, healthcare teams should remain vigilant for postoperative complications.

### Early complications

Myocardial infarction and other cardiac complications are a common cause of death in AAA patients (Powell et al 2017). Cardiac-related deaths occur in 8% of elective EVAR patients and 7.1% of OSR patients, with no significant difference between groups (Paravastu et al 2014). Postoperative ST-segment monitoring should therefore be used in all patients with a high cardiac risk following EVAR as well as all patients after OSR (Chaikof et al 2018). Where this identifies ECG changes, or the patient complains of chest pain, clinicians should order an urgent troponin (Chaikof et al 2018). If sinus tachycardia is identified and/or the patient describes pleuritic chest pain, the differential diagnosis should include a pulmonary embolism (PE) secondary to venous thromboembolism (VTE). For this reason, all AAA patients are assessed for VTE risk and given appropriate thromboprophylaxis (NICE 2020).

In the elective setting, the rate of renal complications requiring postoperative dialysis has been reported as 1.5% following EVAR and 1.2% after OSR, with no statistical difference between methods (Paravastu et al 2014). The 30-day mortality rate in patients who developed renal failure after elective OSR has been reported as 35.0%, compared to 4.3% for those without renal failure (Grant et al 2012). Whereas in patients treated for rAAAs, AKI occurs more frequently following OSR (43%) than EVAR (26%) (Ambler et al 2015). The high rate of AKI after OSR may be attributed to aortic clamping, with more proximal clamp positions during anastomosis resulting in an increased risk of severe AKI (Ambler et al 2015). In comparison, AKI after EVAR is thought to be due to contrast nephropathy (Lee et al 2017). The SVS recommends that at-risk patients undergoing EVAR receive hydration with normal saline or 5% dextrose/sodium bicarbonate, both pre and postoperatively (Chaikof et al 2018).

Abdominal compartment syndrome (ACS) is defined as a sustained intra-abdominal pressure (IAP) >20 mmHg, associated with new organ dysfunction (Kirkpatrick et al 2013). In Sweden, ACS occurs in 1% following OSR and 0.2% after EVAR for unruptured AAAs; however, in those with rAAAs, these figures increased to 3.7% and 7.5%, respectively (Ersryd et al 2019). The reason ACS more commonly occurs in cases of rupture is because the mass effect of bleeding directly increases IAP. This decreases blood flow to the abdominal organs, causing intestinal ischaemia, multiple organ failure and an increased mortality at 30-days after surgery (Ersryd et al 2016, NICE 2020). For these reasons, NICE recommends that patients are assessed for ACS if their condition does not improve postoperatively (NICE 2020). Decompressive laparotomy is the management of choice for ACS (Kirkpatrick et al 2013).

Ischaemic complications are also an important cause of morbidity and mortality following surgical AAA repair, occurring in 9% of patients in the US (Maldonado et al 2004). Of these patients, 75% had lower extremity ischaemia and 14% had colonic ischaemia (CI) (Maldonado et al 2004). CI is associated with especially poor outcomes; mortality rates up to 73% and re-intervention rates of 27–54% have been reported after elective EVAR (Williamson et al 2018). Moreover, EVAR is also associated with a reduced incidence of CI compared with OSR (Lee et al 2016, Williamson et al 2018). However, where OSR is performed, surgeons should preserve blood flow to at least one internal iliac artery to reduce the risk of colonic ischaemia, as well as buttock claudication (Wanhainen et al 2019). The ESVS guidelines emphasize the need to closely monitor all rAAA patients in the postoperative period for CI (Wanhainen et al 2019).

Pulmonary complications are significantly more likely after open repair than EVAR (8.3% vs. 3.1%), and in males, smokers and elderly patients (Paravastu et al 2014, Pasin et al 2017). Other acute complications include multi-organ failure, stroke and incisional hernia (Paravastu et al 2014).

### Late complications

Graft infection is a rare but life-threatening complication of AAA repair. In a large US study, graft infection was reported in 0.19% of OSR patients and 0.16% of EVAR patients at two years post-surgery (Vogel et al 2008). The risk of graft infection is highest in the first year, although it occurs at a median time of 3.0 years (Vogel et al 2008). Emergency procedures and re-operations are particularly associated with graft infections (Tsapralis et al 2011). High-grade infections may result in sepsis, pseudoaneurysm, graft thrombosis, haematemesis due to aortoenteric fistulation and anastomotic disruption (Treska et al 2016). Overall, the mortality rate associated with infection is high, ranging from 37% to 40% depending on the treatment method (Niaz et al 2020).

To prevent graft infection, patients should receive antibiotic prophylaxis before high-risk procedures if the potential for infection exists, or the patient is immunocompromised (Chaikof et al 2018). Where there is a clinical suspicion of infection, investigations should include a computer tomography (CT) scan. If this identifies extensive contamination with gross purulence, extra-anastomotic reconstruction should be performed, followed by excision of all graft material and aortic stump closure (Chaikof et al 2018). In the absence of such contamination, in situ reconstruction with a cryopreserved allograft is preferred (Chaikof et al 2018). The duration of antibiotic treatment after removal of the infected graft is controversial, with some authors recommending life-long treatment and others as little as six weeks (Treska et al 2016).

**Figure 2:**
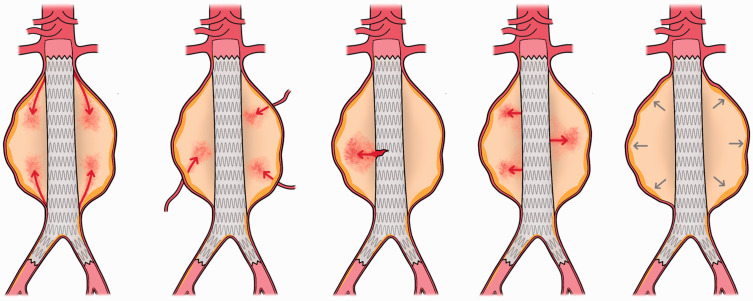
Types of endoleak. (I) Type I: blood flowing into the AAA because of an incomplete/ineffective seal. (II) Type II: retrograde blood flow into the AAA from side branches. (III) Type III: blood flowing into the AAA through endograft defects. (IV) Type IV: blood flowing into the AAA through the stent–graft fabric. (V) Type V: AAA expansion without radiographic evidence of a leak site. Definitions based on NICE 2020

An endoleak is defined as the persistence of blood flow outside an endovascular stent–graft, but within the aneurysm sac (NICE 2020). There are five main types of endoleaks to be aware of (Figure 2). The main danger of endoleaks is secondary rupture of the AAA, which occurs in 2.4% of EVAR patients at a median time of 3.5 years and carries a 30-day mortality rate of 62% (Powell et al 2017). Endoleaks and other EVAR complications like device migration make secondary AAA rupture significantly more prevalent in EVAR patients than OSR patients, whom only experience rupture in 0.1% of cases (Stather et al 2013).

Type II endoleaks are the most common and have an incidence of 10.2% among EVAR patients (Sidloff et al 2013). Fortunately, 35.4% resolve spontaneously (Sidloff et al 2013). However, where resolution does not occur, a persistent type II endoleak increases the risk of AAA growth and rupture (Jones et al 2007). Guidelines therefore recommend surveillance for type II endoleaks not associated with aneurysm expansion and treatment of those associated with aneurysm expansion (Chaikof et al 2018, NICE 2020, Wanhainen et al 2019). One report suggests that interventions are able to successfully seal the endoleak in 71.5% of cases, with translumbar embolization generally producing higher clinical success rates and lower complication rates than transarterial embolization (Sidloff et al 2013).

Type I and III endoleaks are associated with elevated sac pressures and occur in 4.3% and 1.3% of EVAR patients, respectively, accounting for 54% of aneurysm ruptures in EVAR patients (Antoniou et al 2015, Chaikof et al 2018, Powell et al 2017). As such, both NICE and the SVS recommend treatment upon detection using open, endovascular or percutaneous interventions (Chaikof et al 2018, NICE 2020, Wanhainen et al 2019). Interestingly, the majority of proximal type I endoleaks resolve on their own within a year (O’Donnell et al 2018). Clinicians may therefore want to reconsider immediate intervention in these patients. Similarly, type IV endoleaks often seal spontaneously and so intervention is not recommended in this group (Chaikof et al 2018, NICE 2020, Wanhainen et al 2019).

Type V endoleaks (endotension) are rare with newer grafts and occur in only 0.6% of EVAR patients (Powell et al 2017). They are thought to arise secondary to other occult endoleaks, and as the aneurysm diameter enlarges, can lead to more serious endoleaks including type I/III. These patients therefore require thorough investigation (NICE 2020). Treatment should be individualised and may include observation, re-lining of low-porosity endografts or explantation, depending on sac growth (Chaikof et al 2018, Wanhainen et al 2019).

### Surveillance

EVAR patients suffer significantly more complications than OSR patients in the long term; the complication rate has been reported as 10.7 per 100 person-years in EVAR patients, compared with 0.8 per 100 person-years in OSR patients (Powell et al 2017). They are also significantly more likely to require re-interventions, with 26% of EVAR patients needing re-intervention in a 15-year follow-up study, compared with only 12% of OSR patients (Patel et al 2016).

Due to the high incidence of complications in the 5-year period after EVAR, patients should undergo postoperative surveillance (Chaikof et al 2018, NICE 2020, Wanhainen et al 2019). The SVS recommends a contrast-enhanced CT scan (CECT) and a colour duplex ultrasound scan (DUS) within one month (Chaikof et al 2018). In the absence of endoleak or sac enlargement, they recommend another scan at 12 months and yearly follow-up using DUS. If a type II endoleak is found at one month, another CECT and DUS is required at six months. If a stable sac size is observed, then a six-monthly DUS should be carried out for 24 months, followed by yearly follow-up (Chaikof et al 2018).

The ESVS recommend risk stratification based on CECT within 30 days postoperatively (Wanhainen et al 2019). Low-risk patients show no endoleaks, an adequate seal and require CT imaging every five years. Intermediate-risk patients have an adequate seal but a type II endoleak. They require annual follow-up for expansion with a DUS; if there is sac shrinkage ≥1 cm, they are managed as per low-risk patients. If there is expansion ≥1 cm, patients are managed as high risk. High-risk patients have an inadequate seal or type I/III endoleak. These patients should be assessed for re-intervention, especially in type I/III endoleaks. Patients with an inadequate seal but no endoleaks need repeat CECT to accurately assess progression. All patients should be reimaged every five years (Wanhainen et al 2019).

NICE, on the other hand, recommend that the frequency of surveillance should be assessed on a case-by-case basis, depending on each patient’s risk of EVAR-related complications (NICE 2020). They state that CECT or a colour DUS should be used to assess AAA diameter and EVAR device limb kinking, rather than both together. Where endoleak is suspected, CECT should be used for diagnosis. Crucially, a colour DUS alone should not be used due to its suboptimal sensitivity for type I/III endoleaks and dependency on skill (NICE, 2020).

Following OSR, the SVS recommends an abdominal and pelvic CT scan every five years to check for aneurysmal dilatations or anastomotic aneurysms (Chaikof et al 2018). However, NICE do not recommend surveillance post-OSR due to its relatively low incidence of complications (NICE 2020).

## Conclusion

There are multiple surgical methods of treating AAAs, each with different risks and outcomes. The type of operation chosen is therefore complex, and a number of factors need to be considered. Moreover, healthcare professionals should remain alert for common complications that can arise in the postoperative period and patients should undergo surveillance. An awareness of these key perioperative factors is required to optimise the care that these patients receive.

## Key phrases

1. AAAs are associated with significant morbidity and mortality.
2. AAAs can undergo endovascular or open surgical repair.
3. The type of surgical repair chosen depends on a variety of factors.
4. There are many complications that may arise in the postoperative period.

*No competing interests declared.*
